# An eye-tracking method to reveal the link between gazing patterns and pragmatic abilities in high functioning autism spectrum disorders

**DOI:** 10.3389/fnhum.2014.01067

**Published:** 2015-01-14

**Authors:** Ouriel Grynszpan, Jacqueline Nadel

**Affiliations:** ^1^Institut des Systèmes Intelligents et de Robotique (ISIR), Université Pierre et Marie Curie, Centre National de la Recherche ScientifiqueParis, France; ^2^Centre Emotion, Hôpital de La SalpêtrièreParis, France

**Keywords:** eye-tracking, gaze-contingent display, facial expressions, theory of mind, cognition verbs

## Abstract

The present study illustrates the potential advantages of an eye-tracking method for exploring the association between visual scanning of faces and inferences of mental states. Participants watched short videos involving social interactions and had to explain what they had seen. The number of cognition verbs (e.g., think, believe, know) in their answers were counted. Given the possible use of peripheral vision that could confound eye-tracking measures, we added a condition using a gaze-contingent viewing window: the entire visual display is blurred, expect for an area that moves with the participant’s gaze. Eleven typical adults and eleven high functioning adults with Autism Spectrum Disorders (ASD) were recruited. The condition employing the viewing window yielded strong correlations between the average duration of fixations, the ratio of cognition verbs and standard measures of social disabilities.

## Introduction

The rise of affective computing during the last two decades has offered a wide range of possibilities for designing new tools that foster the study of social and emotional impairments. Affective computing is a stream of research that strives to empower computers with abilities to detect, process and respond to social and emotional signals emitted by human users (Picard, 2000). These advances appear to be especially relevant to Autism Spectrum Disorders (ASD) where social interactions represent the core deficit of the syndrome. The last decade has witnessed a steep increase in the number of projects devoted to innovative technologies for evaluating, assisting or training individuals with ASD (Grynszpan et al., 2014). Besides, a promising trend of research relies on the use of eye-tracking to gain a deeper understanding of the cognitive processes that characterize ASD (Boraston and Blakemore, 2007). The exploratory study reported here seeks to present an eye-tracking method for examining social processing in ASD, which takes advantage of real-time eye-based computer interaction.

Social misunderstanding in ASD has been linked to atypical visual scanning patterns (Klin et al., 2002a). Earlier eye-tracking studies used static images to examine the dysfunctional visual exploration of faces attributed to ASD (Pelphrey et al., 2002; van der Geest et al., 2002; Dalton et al., 2005; Corden et al., 2008; Fletcher-Watson et al., 2009). The development of eye-tracking technology has offered new opportunities to investigate gaze through the use of dynamic social scenes, which are closer to real life settings (Klin et al., 2002a). This stream of research has raised hopes that eye-tracking measures would help identify behavioral markers and enable refining the autistic symptomatology. Yet, studies reported inconsistent results: Klin et al.’s (2002b) initial report that adolescents and adults tended to focus more on the mouth and less on the eyes than typical controls has only been partly confirmed (Speer et al., 2007; Norbury et al., 2009). Jones et al. (2008) showed that fixation times on the eyes were reduced in toddlers with ASD, but more recent studies failed to reproduce this finding (Nakano et al., 2010; Chawarska et al., 2013). The most consistent discriminating measure between ASD and typical participants appears to be the fixation times on faces (Riby and Hancock, 2009; von Hofsten et al., 2009; Grynszpan et al., 2012a; Rice et al., 2012; Chawarska et al., 2013; Magrelli et al., 2013). Another promising line of research has been to examine the association between gaze data and standard evaluation instruments of ASD. Klin et al. (2002b) reported that the time spent looking at the mouth region correlated with social abilities in high functioning adolescents and adults. Later research suggested that this correlation depended on the characteristics of the video material, such as the number of characters displayed (Speer et al., 2007) or the space taken on the screen by human faces (Rice et al., 2012). Norbury et al. (2009) failed to reproduce this outcome, but found a negative correlation between the fixation times on the eyes and communicative competencies. The observation that verbally able adults with ASD showed a high association between fixations on facial features and social or communicative abilities has led to the hypothesis that their visual scanning strategies was connected to their linguistic proficiency (Klin et al., 2002b; Norbury et al., 2009).

Studies on linguistic competencies in ASD reveal a primary deficit in pragmatics (Tager-Flusberg, 2000). Grynszpan et al. (2008) suggest that children with ASD experience difficulties in using facial expressions as cues to resolve pragmatic ambiguities in dialogs. Their poor performance in pragmatics have been linked to theory of mind deficiencies (Happé, 1993). Individuals with ASD are reported to produce fewer mental state terms in their narratives (Baron-Cohen et al., 1986). In particular, Tager-Flusberg (2000) underlines their difficulties in using cognition verbs (e.g., think, know, guess) that specifically require theory of mind abilities.

The present article reports an exploratory study that seeks to examine the connections between gaze patterns and communicative competencies. It is part of a larger research program that aims at engineering innovative technologies for investigating gazing patterns in ASD. The method presented in this paper could be applied to various features of social communication, such as language fluency, mental state attribution or emotional expression. Here, we illustrate the potential of this method for revealing associations between gaze patterns and pragmatic competencies. More precisely, the method was used to examine correlations between visual fixations on faces and the production of cognition verbs.

A major pitfall of eye-tracking is that it merely measures focal vision and therefore cannot account for attention that is allotted to the periphery of the visual field. This distinction appears to be especially crucial in ASD, given the clinical reports of individuals with ASD having a greater tendency to rely on peripheral vision (Mottron et al., 2007; Noris et al., 2012). To address this possible confound, when using static stimuli, Spezio et al. (2007) employed the bubble paradigm (Gosselin and Schyns, 2001). In this paradigm, participants are shown series of masked images of a face where only randomly selected portions (bubbles) are visible. The areas of the face that contribute the most to emotion recognition are then computed based on performance obtained with the different masks. This procedure can hardly be directly transposed to dynamic stimuli, as the number of masks would increase exponentially with the number of video frames. We propose an alternative method where the mask is contingent on the gaze orientation: The visible area moves in real time with the focal position of the participant on the screen. This creates a gaze-contingent viewing window that reduces possible reliance on peripheral vision and should hence enhance the congruence between visual attention and focal vision as measured with eye-tracking. In a previous study using this gaze-contingent window, participants with ASD viewed realistic animations of expressive virtual humans (Grynszpan et al., 2012a). In the present study, we sought to test a context closer to real life settings, by showing videos of real life social interactions that participants had to subsequently describe. We computed correlations with cognition verbs production, first, in the normal vision condition and, second, using the gaze-contingent viewing window. Those two conditions were compared in two pilot experiments, one with typical individuals and the other with individuals having ASD. These experiments were meant to provide preliminary observations regarding the potential of the proposed method.

## Method

### Participants

In experiment 1, we tested the feasibility of the method with a normative group of 11 typical adults (3 females 8 males), ranging from 24 to 40 years with a mean age of 31.82 [*SD* = 5.65]. In this pilot study, our goal was to examine the visual strategies employed by the participants who used the gaze-contingent viewing window and not to compare typical controls with individuals having ASD; therefore, in these preliminary experiments, we did not seek to match the normative group of experiment 1 with the ASD group of experiment 2.

Experiment 2 included 11 participants (2 females 9 males) diagnosed with autism by psychiatrists using the DSM-IV R diagnostic criteria. The Autism Diagnostic Interview-Revised (ADI-R; Lord et al., 1994) was used to confirm the diagnosis. Participants’ mean score on the Raven’s Progressive Matrices (Raven and Court, 1986) was 47.03 [*SD* = 10.01]. Their mean Verbal Intelligence Quotient assessed with the Wechsler Adult Intelligence Scale, 3rd edition (Wechsler, 1997), was 88.91 [*SD* = 15.33]. The group was thus considered high functioning. Their age ranged from 17 to 31 with a mean of 21.36 [*SD* = 4.41]. This research was prospectively reviewed and approved by the regional ethics committee of Tours, France. An informed consent was obtained from each participant. In addition, parents’ consents were obtained for minor participants.

### Procedure

**Figure 1:**
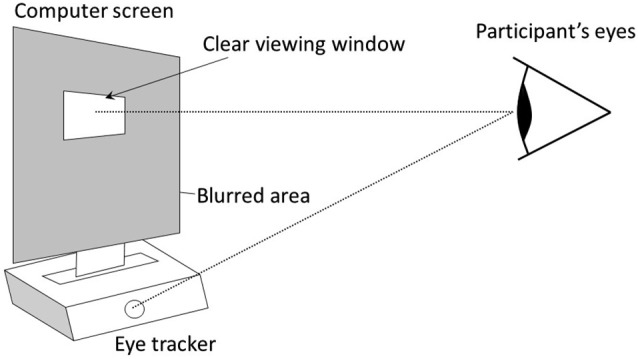
**The graphic display is entirely blurred except for a window centered on the focal point of the participant, which is detected in real time using an eye-tracker (model EYE-TRAC 6 Desktop from Applied Science Laboratories, 50 Hz sampling rate)**. The eye-tracker remotely measured gaze orientation, without constraining head movements or requiring a helmet.

**Figure 2:**
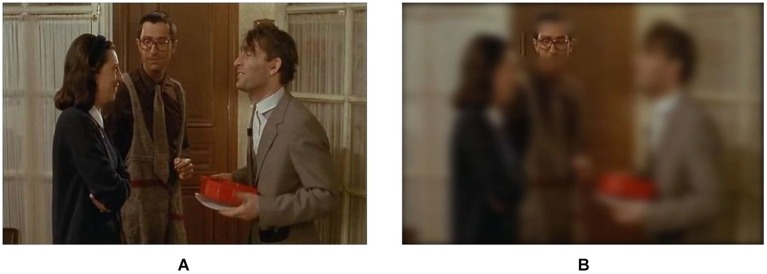
**Sample screen shots of an extract of the movie “Le père Noël est une ordure” (directed by Jean-Marie Poiré in 1982) that was used as a stimulus (A) in the normal vision condition; (B) in the gaze-contingent viewing window condition**.

The same design was used in experiment 1 and 2. It was composed of an initial normal vision condition followed by a condition using the gaze-contingent viewing window. In the two conditions, participants watched a 2 min video on a 19 inches computer screen that was positioned above a remote eye-tracker (model EYE-TRAC 6 Desktop from Applied Science Laboratories). In the gaze-contingent viewing window condition, the entire graphic display was blurred (using Gaussian smoothing), except for a window centered on the focal point of the participant that moved in real time with her/his gaze (Figures 1, 2). This viewing window was a rectangle with rounded angles measuring 200 × 80 pixels, which amounted to visual angles of 6° × 2° 22′, therefore covering the fovea visual region in the horizontal direction. The size of this window was determined so that it could at least encompass the two eyes of any face shown in the video. Further technical details are available in Grynszpan et al. (2012a). Two videos were used, one in each condition. They were randomly counterbalanced across participants. Both were movie extracts displaying a social interaction where two protagonists were acting hypocritically toward a third one. Their behavior was contradicting their speech, thus yielding a comical effect. Visual attention to facial expressions was essential for understanding these two movie extracts. In one video^1^, a woman and a man were greeting a neighbor and praising the dish that he prepared, yet their faces and attitudes clearly showed that they were disgusted. In the other video^2^, two men were lauding the dance performance of a woman, although their non-verbal behaviors showed contempt.

After each movie extract, participants were asked to describe what had happened. Their answers were recorded and analyzed by two independent judges who computed the ratio of cognition verbs (e.g., think, believe, know). To calculate this ratio, the number of cognition verbs in each participant’s answer was divided by the total number of verbs employed. The concordance correlation coefficient (Lin, 1989) between judges was *ρ_c_* = 0.91. To analyze eye-tracking data, rectangular Areas Of Interest (AOI) were defined around the faces of the protagonists. We used a software prototype developed for a previous study (Grynszpan et al., 2012a) to adjust the position of the AOI on video frames so that they would remain centered on the faces throughout the movie extracts. A proprietary algorithm of Applied Science Laboratories was used to compute fixations on the basis of clusters of Points-Of-Gaze (POG) remaining for at least 100 ms in 1° of visual angle. Two gaze variables are considered here: the total fixation time on faces, that is, the sum of the fixation durations; and the average fixation duration on faces. In experiment 2, the ADI-R Reciprocal Social Interaction sub-scores and the Childhood Autism Rating Scale (CARS; Schopler et al., 1980) were used as measures of social disability. Although the CARS was originally meant for children, it is also recommended for use in adults (Ozonoff et al., 2005). The ADI-R and the CARS are instruments designed to assist clinicians in diagnosing ASD. The ADI-R is a semi-structured interview of parents or caregivers that is used to retrieve information on the current behavior and developmental history of the individual. The CARS is a rating scale based on the observation of the individual’s behavior.

## Results

The data analyses were carried out with Statistica software.^3^ For the two experiments, we first verified whether the introduction of the gaze-contingent viewing window altered visual exploration and narrative performance by calculating Student’s *t*-tests that compared the two viewing conditions. We then computed Pearson’s correlation coefficients between the ratio of cognition verbs and the gaze variables.

In experiment 1, the data of the normative group showed differences in visual patterns between the two viewing conditions. The total fixation time on faces was higher in the gaze-contingent viewing window condition than in the normal vision condition [*t*_(10)_ = 2.33, *p* = 0.042]. The average duration of fixations on faces was also higher in the gaze-contingent viewing window condition compared with the normal vision condition [*t*_(10)_ = 5.70, *p* < 0.001]. The ratio of cognition verbs did not differ significantly between the two viewing conditions. We did not find any correlations between any of these three variables. Indeed, the ratio of cognition verbs did not correlate with the total fixation time on faces [normal vision condition: *r* = 0.13 *p* = 0.71; gaze-contingent viewing window condition: *r* = 0.40 *p* = 0.23], nor did it with the average fixation duration [normal vision condition: *r* = 0.01 *p* = 0.98; gaze-contingent viewing window condition: *r* = 0.27 *p* = 0.42].

In experiment 2, no significant differences were found between the two viewing conditions on any of the measures, that is, the ratio of cognition verbs [*t*_(10)_ = 0.41, *p* = 0.69], the total fixation time on faces [*t*_(10)_ = 0.76, *p* = 0.46] and the average fixation duration on faces [*t*_(10)_ = 0.48, *p* = 0.64]. Contrasting with the normative group in experiment 1, the total fixation time on faces correlated with the ratio of cognition verbs in the gaze-contingent viewing window condition [*r* = 0.70, *p* = 0.017]. The average fixation duration on faces correlated with the ratio of cognition verbs in the normal vision condition [*r* = 0.60, *p* = 0.05] and in the gaze-contingent viewing window condition [*r* = 0.87, *p* < 0.001]. In the latter condition, the ratio of cognition verbs correlated negatively with the CARS scores [*r* = −0.66, *p* = 0.03] and the average duration of fixations on faces correlated negatively with CARS scores [*r* = −0.76, *p* = 0.006] and with the ADI-R sub-scores in the Reciprocal Social Interaction domain [*r* = −0.65, *p* = 0.031].

## Discussion

Studies using eye-tracking techniques to analyze gaze exploration in ASD have been mostly involved in the description of facial regions of interests and their link with social impairments. The goal of the present study was to pinpoint more precise components of social impairments, by exploring the potential relationship between gaze exploration of faces and inference of mental states in others. Indeed, inferring mental states is often facilitated by deciphering facial expressions. Cognition verbs were used here as indices of mental states processing. Results of experiment 2 show a positive correlation between gaze variables and cognition verbs in participants with ASD. In other words, the more they were attentive to the dynamics of the facial expressions, the more they would use cognition verbs as if the latter were directly derived from the former. This suggests that their mentalistic insight depends predominantly on a face reading strategy whereby mental states are mapped onto behavioral changes perceived on the face. Interestingly, the severity of autism, as assessed by the CARS and the ADI-R sub-scores in the Reciprocal Social Interaction domain, appeared to be related to poor gaze exploration of faces and poor production of mental state terms.

In experiment 1, the normative group of typical participants modulated their visual behavior to adapt to the gaze-contingent viewing window by increasing their fixation durations on faces. This is consistent with our previous findings (Grynszpan et al., 2012b). The reason why fixation durations on faces did not correlate with the ratio of cognitive verbs for typical participants could conceivably be explained by the fact that their social insight is not solely dependent on facial expressions. In experiment 2, we did not observe an adaptive change in the visual exploration strategies of the ASD group. Although we refrained from comparing groups here, such an alteration in ASD of the typical modulation effect on gaze patterns induced by the viewing window has already been addressed in a previous publication (Grynszpan et al., 2012a). The lack of change in fixation times on faces for ASD participants suggests that they relied on visual strategies when using the gaze-contingent viewing window that were similar to those used in the normal viewing condition. The outcomes derived from the gaze-contingent viewing window should therefore be indicative of their visual exploration in more natural settings.

Recent studies have attempted to tackle discrepancies found in eye-tracking experiments, by devising sophisticated analyses based on the distance between the gaze position of ASD participants and typical gaze patterns (Nakano et al., 2010) or pre-defined targets in the video material (Falck-Ytter et al., 2013). To our knowledge, none of these approaches took into account the possible bias of peripheral vision. Despite anecdotal accounts that individuals with ASD tend to rely more frequently on their peripheral vision than typical peers, especially during social interactions (Bogdashina, 2003; Williams, 2007), there has been relatively little research exploring this issue (Mottron et al., 2007; Grubb et al., 2013; Milne et al., 2013). Most eye-tracking studies focus on central vision, based on the assumption that it matches visual attention. However, detecting that the focal point of gaze is on the mouth does not prevent attention from being directed to the eyes and vice-versa. Such discrepancies could be even more critical in ASD, given the accounts of peculiar use of peripheral vision. The gaze-contingent viewing window method proposed here is meant to reduce this possible confound and indeed, our results showed strong significant correlations with measures of social disability for fixations on the face without having to distinguish between the eyes and mouth regions.

Most eye-tracking studies used the total fixation time on a given AOI as the main outcome measure (Boraston and Blakemore, 2007). Although the assumption that fixation times on a given detail can be summed makes sense for static stimuli, it is less obvious for dynamic stimuli where the information conveyed by the face changes from one fixation to another. The average fixation duration seems more relevant in this regard as it yields an indication of how long a continuous expressive facial motion is attended to by the participant. In experiment 2, this latter measure was strongly associated with the production of cognition verbs in the normal vision condition and when the viewing window was used.

It should be noted that even with the limited sample of participants with ASD used in experiment 2, the method we propose revealed strong significant associations between mental state attribution abilities, social competence and visual scanning of faces. The participants in our sample were all adults on the higher range of IQ scores. So the present findings cannot be generalized to the entire spectrum. The gaze-contingent viewing window method could reveal alternative strategies when applied to different sub-groups across the spectrum, shedding light on their distinctive cognitive functioning.

The gaze-contingent system that we presented here seems useful to examine visual exploration of social scenes. However, the role of gaze in social communication extends beyond purely perceptual functions. It can also assume an active role in face-to-face interactions as for instance in joint attention situations, where it can be used to orient a partner’s attention towards an object of interest (Emery, 2000). The development course of ASD is considered to be strongly associated with impairments in joint attention (Charman, 2003). Gaze-contingent displays could be employed to study joint attention. We designed a platform that displays a virtual human character whose gaze orientation is controlled by an eye-tracking device (Courgeon et al., 2014). It can thus be used to simulate joint attention in systematic and controlled conditions that approach naturalistic situations. Our future work will seek to evaluate the potential of this platform for the study of joint attention in the typical population and for individuals with ASD.

## Conflict of interest statement

The authors declare that the research was conducted in the absence of any commercial or financial relationships that could be construed as a potential conflict of interest.

## Footnotes

^1^Taken from the film “Le père Noël est une ordure”, directed by Jean-Marie Poiré in 1982.

^2^Taken from the movie “Podium”, directed by Yann Moix in 2004.

^3^www.statsoft.com
